# Supplementary figures and images for: Cardiovascular Disease and Diabetes Are Among the Main Underlying Causes of Death in Twenty Healthcare Facilities Across Two Cities in the Democratic Republic of Congo
Source: Int J Environ Res Public Health. 2024 Oct 31;21(11):1450. doi: 10.3390/ijerph21111450 (PMC11593621; doi:10.3390/ijerph21111450)

## Supplementary data

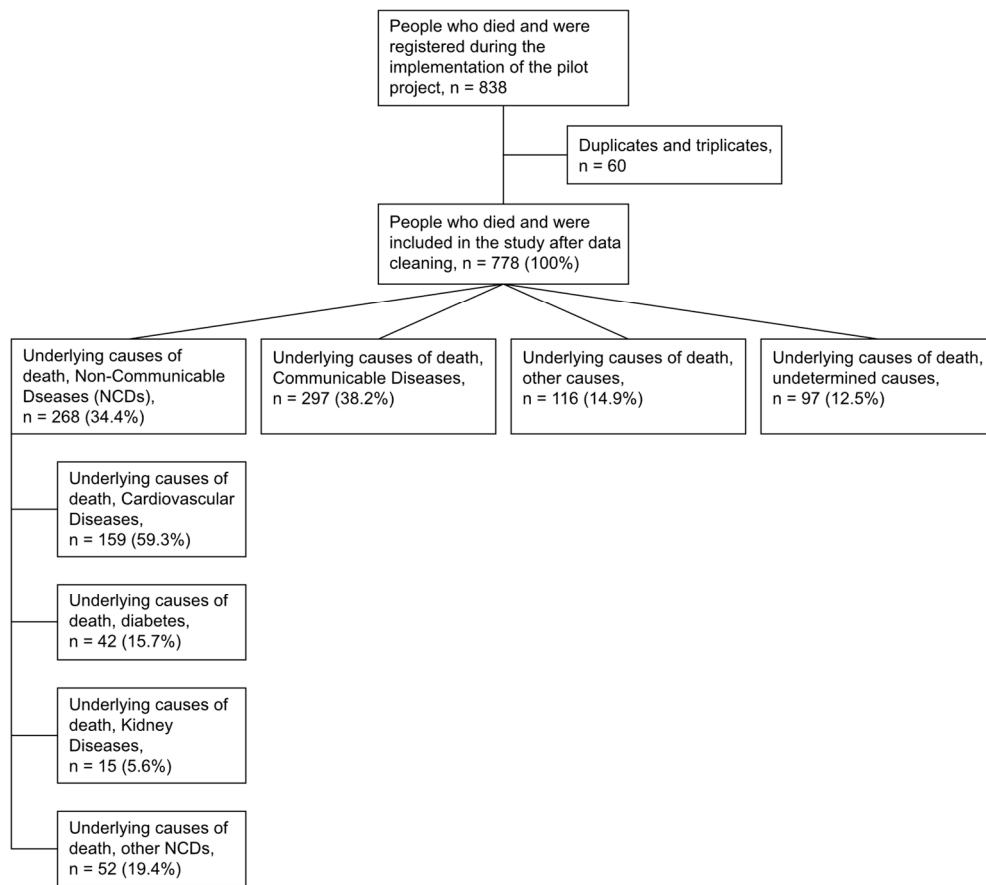

**Figure S1.** Classification of database subjects.

Supplement: Supplementary file 1 [file ijerph-21-01450-s001.zip › ijerph-3234274-supplementary.pdf]
